# Baseline characteristics and comorbidities in the CAnadian REgistry for Pulmonary Fibrosis

**DOI:** 10.1186/s12890-019-0986-4

**Published:** 2019-11-27

**Authors:** J. H. Fisher, M. Kolb, M. Algamdi, J. Morisset, K. A. Johannson, S. Shapera, P. Wilcox, T. To, M. Sadatsafavi, H. Manganas, N. Khalil, N. Hambly, A. J. Halayko, A. S. Gershon, C. D. Fell, G. Cox, C. J. Ryerson

**Affiliations:** 1 Department of Medicine, University of Toronto, Toronto, Canada; 2 University Health Network, 9N-945 585 University Avenue, Toronto, M5G 2N2 Canada; 3 Firestone Institute for Respiratory Health, Department of Medicine, McMaster University, Hamilton, Canada; 4 Department of Pulmonary and Critical Care Medicine, King Fahad Medical City, Riyadh, Saudi Arabia; 5 Département de Médecine, Centre Hospitalier de l’Université de Montréal, Montreal, Canada; 6 Department of Medicine, University of Calgary, Calgary, Canada; 7 Department of Medicine, University of British Columbia, Vancouver, Canada; 8 Centre for Heart Lung Innovation, University of British Columbia, Vancouver, Canada; 9 Institute for Clinical Evaluative Sciences, Toronto, Canada; 10 Child Health Evaluative Sciences, The Hospital for Sick Children, Toronto, Canada; 11 Dalla Lana School of Public Health, University of Toronto, Toronto, Canada; 12 Institute for Heart and Lung Health, Department of Medicine, University of British Columbia, Vancouver, Canada; 13 Faculty of Pharmaceutical Sciences, University of British Columbia, Vancouver, Canada; 14 Department of Internal Medicine, University of Manitoba, Winnipeg, Canada

**Keywords:** Registries, Lung diseases, Interstitial, Idiopathic pulmonary fibrosis, Comorbidity, Environmental exposure

## Abstract

**Background:**

The CAnadian REgistry for Pulmonary Fibrosis (CARE-PF) is a multi-center, prospective registry designed to study the natural history of fibrotic interstitial lung disease (ILD) in adults. The aim of this cross-sectional sub-study was to describe the baseline characteristics, risk factors, and comorbidities of patients enrolled in CARE-PF to date.

**Methods:**

Patients completed study questionnaires and clinical measurements at enrollment and each follow-up visit. Environmental exposures were assessed by patient self-report and comorbidities by the Charlson Comorbidity Index (CCI). Baseline characteristics, exposures, and comorbidities were described for the overall study population and for incident cases, and were compared across ILD subtypes.

**Results:**

The full cohort included 1285 patients with ILD (961 incident cases (74.8%)). Diagnoses included connective tissue disease-associated ILD (33.3%), idiopathic pulmonary fibrosis (IPF) (24.7%), unclassifiable ILD (22.3%), chronic hypersensitivity pneumonitis (HP) (7.5%), sarcoidosis (3.2%), non-IPF idiopathic interstitial pneumonias (3.0%, including idiopathic nonspecific interstitial pneumonia (NSIP) in 0.9%), and other ILDs (6.0%). Patient-reported exposures were most frequent amongst chronic HP, but common across all ILD subtypes. The CCI was ≤2 in 81% of patients, with a narrow distribution and range of values.

**Conclusions:**

CTD-ILD, IPF, and unclassifiable ILD made up 80% of ILD diagnoses at ILD referral centers in Canada, while idiopathic NSIP was rare when adhering to recommended diagnostic criteria. CCI had a very narrow distribution across our cohort suggesting it may be a poor discriminator in assessing the impact of comorbidities on patients with ILD.

## Background

Fibrotic interstitial lung diseases (ILDs) encompass a wide variety of disorders that can be related to underlying connective tissue disease (CTD), occupational or environmental exposures, or an unknown cause [1]. Commonly reported fibrotic ILD subtypes include idiopathic pulmonary fibrosis (IPF), CTD-associated ILD (CTD-ILD), idiopathic non-specific interstitial pneumonia (NSIP), and chronic hypersensitivity pneumonitis (HP). Limited epidemiologic data are available for fibrotic ILDs other than IPF, and the heterogeneity and relative rarity of these ILD subtypes are significant barriers to research.

Comorbid conditions are increasingly described amongst ILD patients. Data suggest an increased prevalence of several comorbidities in patients with IPF compared to the general population [2]. These comorbidities contribute to the high economic burden, substantial morbidity, and early mortality of IPF. Recent studies have described comorbidities in international cohorts of patients with IPF [3–6]; however, there are minimal data for non-IPF fibrotic ILDs.

The CAnadian REgistry for Pulmonary Fibrosis (CARE-PF) is a multi-center, prospective registry designed to study the natural history of fibrotic ILD in a Canadian population [7]. This is the largest cohort of ILD patients in Canada and among the largest internationally, with the goal of providing valuable insight into the characteristics, management, and outcomes of these relatively rare diseases. The aim of this sub-study was to describe the baseline characteristics, risk factors, and comorbidities of patients enrolled in CARE-PF during the first 18 months of patient recruitment.

## Methods

### Study design

A cross-sectional study of patients enrolled in the first 18 months (January 2016 to July 2017) of CARE-PF was performed. As previously described [7], CARE-PF is an open-ended prospective cohort of Canadian patients with fibrotic ILD of any subtype who are 18 years or older, able to provide informed consent and complete questionnaires in English or French. There are no exclusion criteria. Patients are recruited from 6 specialized ILD clinics; 2 at the University of British Columbia (Vancouver, BC), and 1 at each of the University of Calgary (Calgary, AB), McMaster University (Hamilton, ON), University of Toronto (Toronto, ON), and University of Montreal (Montreal, QC). Ethics approval for this study was obtained by the research ethics boards at each participating site.

All participating CARE-PF sites are ILD referral centers and were initially selected based on meeting the following criteria: (1) access to a formal multidisciplinary discussion, (2) access to a respirologist with ILD expertise and (3) access to the appropriate research infrastructure. Each site holds regular multidisciplinary conferences with ILD clinicians, chest radiologists, and lung pathologists to establish diagnoses using a standardized approach across all registry centers based on established guidelines where available. IPF was diagnosed according to established guideline criteria [8, 9]. Probable IPF was defined as a multidisciplinary diagnosis of IPF that did not meet guideline criteria for a definite diagnosis, consistent with recently published criteria [9, 10]. Given the absence of established criteria, a diagnosis of chronic HP was defined by the presence of lung fibrosis and HP being the leading diagnosis on at least two out of three domains (clinical, radiological, pathological) [11]. A diagnosis of idiopathic NSIP required confirmation by surgical lung biopsy (SLB) as previously described [12]. Patients without a confident diagnosis (< 50% confidence) were considered to have unclassifiable ILD in accordance with the ontologic framework suggested by an international working group [13]. CARE-PF identifies those patients that meet the proposed research criteria for interstitial pneumonia with autoimmune features (IPAF) after rheumatologic assessment; however, this is not currently an accepted clinical diagnosis and such patients were considered to have unclassifiable ILD in this study [14].

### Measurements

Patients complete study questionnaires and clinical measurements at enrollment and each follow-up visit. The baseline study questionnaire records demographics (age, date of birth, ethnicity, and race), smoking history and pack-years, family history of pulmonary fibrosis (biological parent, sibling, or child), regular or repeated environmental exposures, occupational or hobby exposures, medications and medical history (full list of exposures available in Additional file 1: Table S1). Shortness of breath, cough and quality of life questionnaires are completed at baseline and each follow-up visit. Comorbidities are collected both by patient report in the baseline and follow-up questionnaires and by study team members using the patient’s medical record to confirm the presence or absence of all comorbidities included in the Charlson Comorbidity Index (CCI; Additional file 2: Table S2). CCI is a validated tool using patient comorbidities to predict 1-year mortality in adults [15]. Nineteen comorbidity categories are assigned a weighted score based on the relative risk of 1-year mortality. Pulmonary function tests were performed according to standard techniques [16–18]. Additional details regarding CARE-PF study questionnaires and methods have been previously published [7].

### Statistical analysis

Analyses were primarily descriptive. Baseline characteristics, exposures, and comorbidities were described for the overall study population, incident cases and the subsets of patients with IPF, non-IPF idiopathic interstitial pneumonia (IIP), CTD-ILD, chronic HP, unclassifiable ILD, sarcoidosis, and other ILDs. Between group differences were assessed using Chi-square or Wilcoxon Rank Sum, as appropriate. A *p*-value of < 0.05 is used to indicate statistical significance. Analyses were performed using SAS software, version 9.4 (SAS Institute Inc., NC).

## Results

### Baseline characteristics and ILD diagnosis

**Fig. 1:**
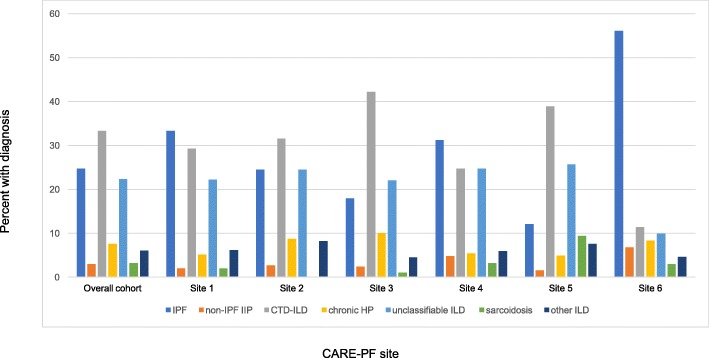
Percent ILD diagnosis overall and by CARE-PF site. Other ILD includes: Vasculitis, diffuse alveolar hemorrhage, drug related, pneumoconiosis, post-acute respiratory disease syndrome, aspiration, eosinophilic pneumonia, pleuroparenchymal fibroelastosis, lymphangioleiomyomatosis, Langerhan’s cell histiocytosis, neuroendocrine cell hyperplasia, pulmonary alveolar proteinosis. Abbreviations: CTD-ILD, connective tissue disease-associated ILD; HP, hypersensitivity pneumonitis; IIP, idiopathic interstitial pneumonia; ILD, interstitial lung disease; IPF, idiopathic pulmonary fibrosis

The full cohort included 1285 patients with ILD (961 incident cases (74.8%) and 324 prevalent cases (25.2%)). Diagnoses included IPF (24.7%), non-IPF IIP (3.0%), CTD-ILD (33.3%), chronic HP (7.5%), unclassifiable ILD (22.3%), sarcoidosis (3.2%), and other ILDs (6.0%). There was some variation in the percentage of ILD subtype from each center (Fig. 1). Of the 317 patients with IPF, 210 (66.2%) had a definite diagnosis and 107 (33.8%) had a probable diagnosis following multidisciplinary discussion. Of the 286 patients with unclassifiable ILD, the most frequently listed differential diagnoses were 53 IPF (27.6%), 36 chronic HP (18.8%), and 33 idiopathic NSIP (17.8%). Criteria for IPAF were met in 47 patients with unclassifiable ILD, representing 16% of this population and 4% of the full cohort. The proportion of each diagnosis was similar comparing incident and prevalent cases, although CTD-ILD was more common amongst prevalent cases (Additional file 3: Table S3).

Baseline characteristics are shown in Table 1 for the full cohort and in Additional file 4: Table S4 for incident cases. Most patients were older adults with similar proportions of men and women, and on average had mild and moderate reduction in FVC and DLCO, respectively. Compared to other diagnoses, patients with IPF and unclassifiable ILD were older, patients with IPF were more frequently male, and patients with sarcoidosis had higher lung function. Oxygen use was reported in 22.1% of the cohort, ranging from 5.3% of those with sarcoidosis to 34.1% of patients with IPF. Surgical lung biopsy was performed in 270 patients (21.0%), with 24.1% of biopsied patients remaining unclassifiable following multidisciplinary review. The proportion of unclassifiable ILD among biopsied patients was similar across most sites.

**Table 1:** Baseline characteristics in the full cohort and stratified by ILD diagnosis^a^

Characteristic	Full cohort *n* = 1285	IPF *n* = 317	Non-IPF IIP *n* = 39	HP *n* = 97	Sarcoid *n* = 41	CTD-ILD *n* = 428	Unclassifiable *n* = 286	Other ILD *n* = 77
Age, years	64.8+/− 11.8	70.9+/−8.5	61.7+/− 11.2	63.7+/− 10.2	55.6+/− 11.5	60.3+/− 11.9	67.6+/− 10.5	62.0+/− 14.7
Male sex	633 (49.3)	229 (72.2)	19 (48.7)	43 (44.3)	21 (51.2)	130 (30.4)	150 (52.5)	41 (53.3)
Ever smoked	781 (62.9)	240 (78.2)	29 (76.3)	55 (57.9)	19 (47.5)	221 (53.8%)	177 (64.4)	40 (52.6)
Pack-years	20.5 (8.3, 36.0)	25.0 (14.3,40.0)	21.3 (11.1, 31.0)	16.1 (7.0, 35.1)	7.0 (2.7, 11.4)	15.0 (4.7, 30.0)	22.0 (10, 37.5)	27.1 (5.3, 52.5)
BMI, kg/m^2^	28.7+/−5.8	28.7+/−4.9	29.3+/−6.5	30.5+/−6.2	29.0+/− 6.3	27.1+/− 5.7	30.3+/− 5.8	28.9+/− 6.4
FVC, %-predicted	74.5+/−20.3	72.8+/− 19.5	72.1+/− 18.3	67.7+/− 21.0	86.9+/−16.0	74.5+/−19.9	76.0+/− 20.7	79.9+/−22.9
DLCO, %-predicted	56.7+/−20.1	49.9+/−16.7	52.4+/−20.4	54.0+/−19.0	80.1+/− 18.4	56.4+/− 19.2	59.2+/− 19.5	68.7+/−25.7
Oxygen use	259 (22.1)	100 (34.1)	11 (29.0)	27 (31.0)	2 (5.3)	59 (15.3)	45 (17.4)	15 (21.1)
SLB	270 (21.0)	48 (15.1)	23 (59.0)	50 (51.6)	16 (39.0)	52 (12.2)	65 (22.7)	16 (20.8)
CCI	1.7+/−1.3	1.3+/−1.2	1.2+/− 0.9	1.3+/− 1.0	1.4+/− 1.4	2.1+/− 1.0	1.7+/− 1.6	1.7+/−1.4

Other ILD includes: Vasculitis, diffuse alveolar hemorrhage, drug related, pneumoconiosis, post-acute respiratory disease syndrome, aspiration, eosinophilic pneumonia, pleuroparenchymal fibroelastosis, lymphangioleiomyomatosis, Langehan’s cell histiocytosis, neuroendocrine cell hyperplasia, pulmonary alveolar proteinosis

*Abbreviations*: *BMI* Body mass index, *CCI* Charlson Comorbidity Index, *CTD-ILD* Connective tissue disease-associated ILD, *DLCO* Diffusing capacity of the lung for carbon monoxide, *FVC* Forced vital capacity, *HP* Hypersensitivity pneumonitis, *IIP* Idiopathic interstitial pneumonia, *ILD* Interstitial lung disease, *IPF* Idiopathic pulmonary fibrosis, *SLB* Surgical lung biopsy

^a^ Data shown are mean+/−standard deviation, median (interquartile range) or number (percent)

### Environmental exposures and risk factors

**Fig. 2:**
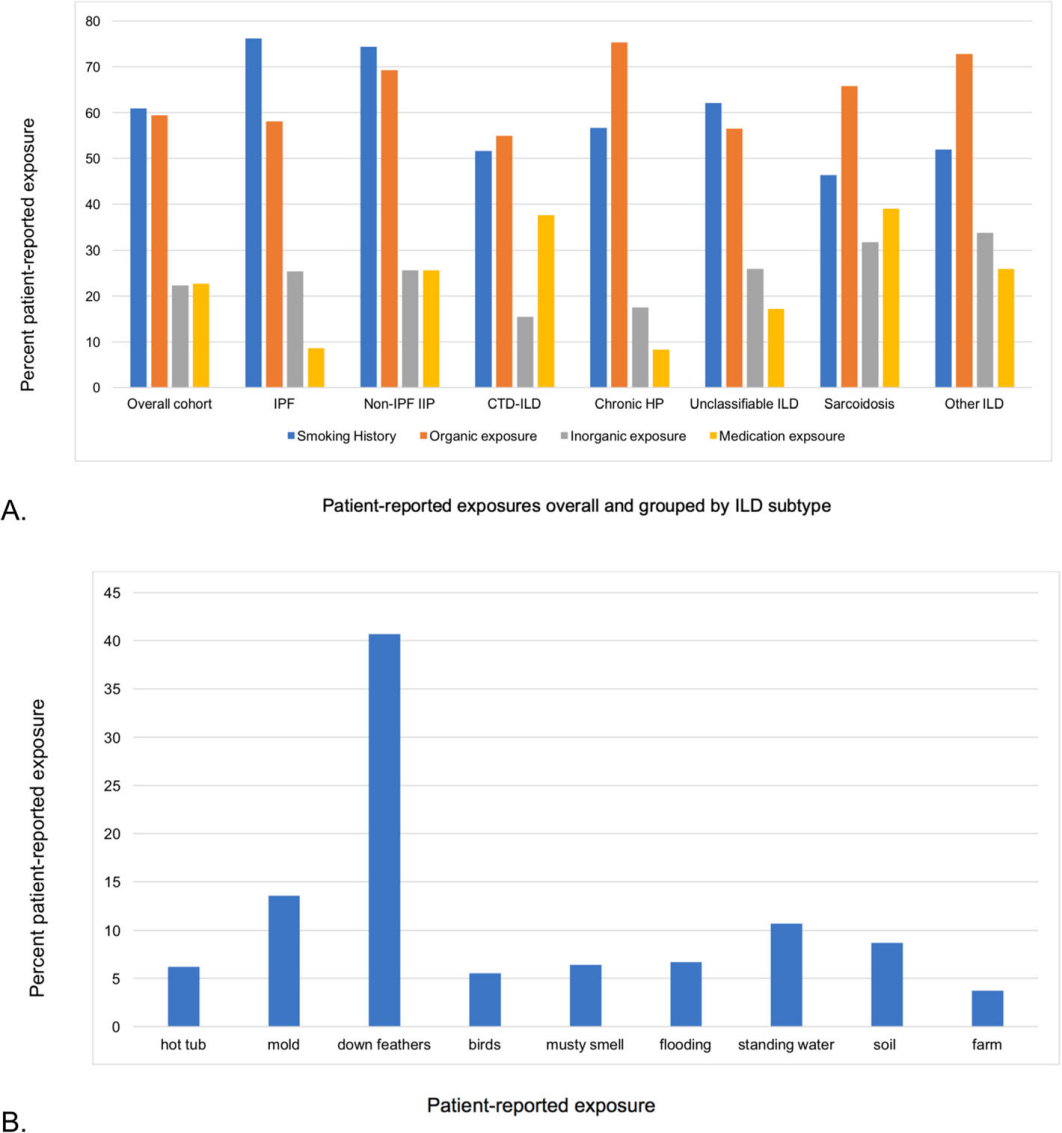
**a** Patient-reported exposure overall and by ILD subtype. Other ILD includes: Vasculitis, diffuse alveolar hemorrhage, drug related, pneumoconiosis, post-acute respiratory disease syndrome, aspiration, eosinophilic pneumonia, pleuroparenchymal fibroelastosis, lymphangioleiomyomatosis, Langerhan’s cell histiocytosis, neuroendocrine cell hyperplasia, pulmonary alveolar proteinosis. Abbreviations: CTD-ILD, connective tissue disease-associated ILD; HP, hypersensitivity pneumonitis; IIP, idiopathic interstitial pneumonia; ILD, interstitial lung disease; IPF, idiopathic pulmonary fibrosis. **b** Patient-reported organic exposures for the overall cohort

Patient-reported environmental exposures stratified by ILD subtype are shown in Fig. 2a. The most commonly reported organic exposures in the overall study population were down feathers (40.7%), mold (13.6%) and water (10.7%) (Fig. 2b). Organic exposures were frequently reported in all ILD subtypes, but most commonly reported by those with chronic HP (75.3% versus 59.4% in the overall cohort). Mold, a musty smell, flooding, and soil exposure were twice as frequently reported in those with chronic HP as compared to other diagnoses, and exposure to birds and farming were 5 and 3 times more frequently reported, respectively. Hot tub and down feather exposure were similarly reported in patients with and without chronic HP and exposure to standing (pooled) water was almost twice as frequently reported in patients with a diagnosis other than chronic HP. Inorganic exposures were reported in 22.3% of the cohort, and most commonly reported amongst other ILDs, of which asbestos accounted for the largest proportion. The most frequently reported occupations with a risk of inorganic exposure were welder (6.6%), plumber (5.5%) and cement worker (5.5%). Patient-reported exposures were similar in IPF patients with a definite versus probable diagnosis. Patient-reported family history of ILD was 12.0% in the overall cohort and more common in patients with IPF (17.4%) and non-IPF IIPs (15.8%) compared to the remainder (10.0%).

### Comorbidities

**Fig. 3:**
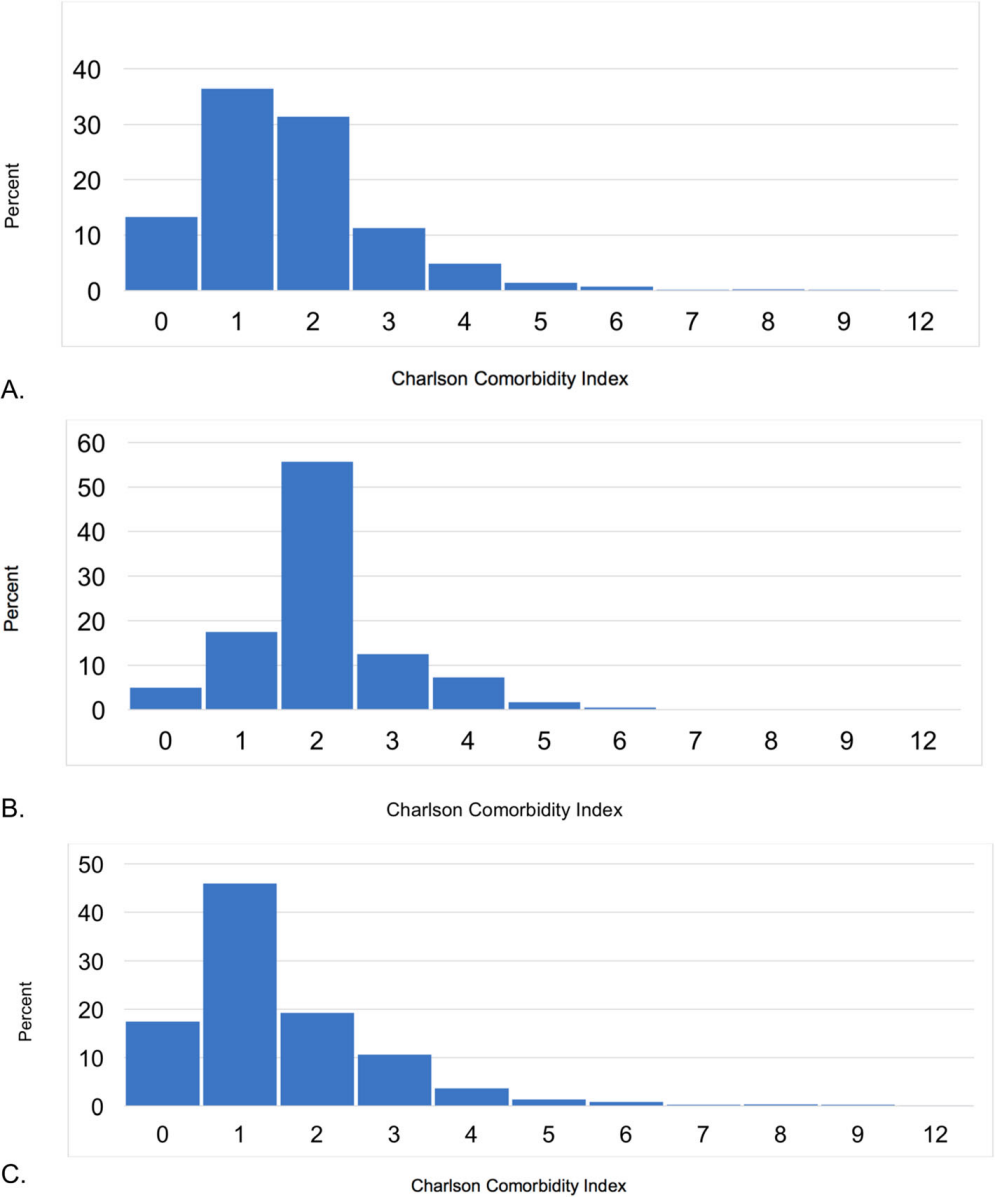
**a** Distribution of Charlson Comorbidity Index for the full cohort. **b** Distribution of Charlson Comorbidity Index for CTD-ILD. Abbreviations: CTD-ILD, connective tissue disease-interstitial lung disease. **c** Distribution of Charlson Comorbidity Index for non-CTD-ILD. Abbreviations: CTD-ILD, connective tissue disease-interstitial lung disease

**Fig. 4:**
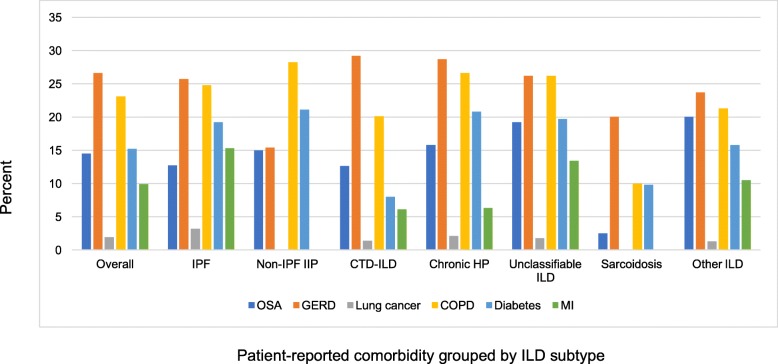
Patient-reported comorbidities by ILD subtype. Other ILD includes: Vasculitis, diffuse alveolar hemorrhage, drug related, pneumoconiosis, post-acute respiratory disease syndrome, aspiration, eosinophilic pneumonia, pleuroparenchymal fibroelastosis, lymphangioleiomyomatosis, Langerhan’s cell histiocytosis, neuroendocrine cell hyperplasia, pulmonary alveolar proteinosis. Abbreviations: COPD, chronic obstructive pulmonary disease; CTD-ILD, connective tissue disease-associated ILD; GERD, gastroesophageal reflux disease; HP, hypersensitivity pneumonitis; IIP, idiopathic interstitial pneumonia; ILD, interstitial lung disease; IPF, idiopathic pulmonary fibrosis; MI, myocardial infarction; OSA, obstructive sleep apnea

The CCI was similar across ILDs and ranged from a mean of 1.2+/− 0.9 in non-IPF IIPs to 2.1+/− 1.0 in CTD. Overall mean (SD) and median (IQR) CCI were 1.7+/− 1.3 and 2 (1, 2), respectively. After excluding those with CTD, mean and median CCI decreased to 1.5+/− 1.4 and 1 (1, 2), respectively. The distribution of the CCI was limited, with 81.1% having a mean score ≤ 2 and an IQR of only 1 (Fig. 3a-c). The most common patient-reported comorbidities were gastroesophageal reflux disease (GERD) (26.6%), chronic obstructive pulmonary disease (COPD) (23.1%), diabetes (15.2%), and obstructive sleep apnea (OSA) (14.5%). GERD was most frequently reported in CTD-ILD, COPD and diabetes were most frequently reported in non-IPF IIPs, and OSA was most frequently reported in unclassifiable ILD (Fig. 4). Of note GERD and OSA are not captured in the CCI. CCI did not appear to increase with decreasing FVC (*p* = 0.05).

## Discussion

We described the baseline characteristics, risk factors, and comorbidities of patients with fibrotic ILD enrolled during the first 18 months of CARE-PF, a multicenter prospective registry inclusive of all fibrotic ILD subtypes that is amongst the largest registries of its kind. CTD-ILD, IPF, and unclassifiable ILD made up 80% of ILD diagnoses at ILD referral centers in Canada, while idiopathic NSIP was rare when adhering to previously recommended diagnostic criteria. Comorbidities were frequent, with limited ability of the CCI to represent the extent and spectrum of comorbid diseases in this population.

Prospective registries can provide insight into ‘real world’ disease epidemiology, natural history, treatments, and outcomes. They are particularly useful when studying heterogeneous and rare diseases such as fibrotic ILDs; however, many registries have focused only on IPF with limited data for other fibrotic ILD subtypes. In our registry, CTD-ILD was the most frequent diagnosis (33.3%), followed by IPF (24.7%) and unclassifiable ILD (22.3%). In contrast, a recently published large prospective ILD registry from India found HP was the most common ILD subtype (47.3%), followed by a much lower frequency of CTD-ILD (13.9%) and IPF (13.7%) [19]. Older epidemiologic data describe similarly variable frequencies of ILD diagnoses. This wide variability is likely multifactorial, related to clinical setting and study design, genetic and geographic heterogeneity, populations with variable risk factors, differences in diagnostic approach, and changes in diagnostic criteria over time.

We report a higher percentage of unclassifiable ILD and a lower percentage of idiopathic NSIP compared to many other ILD cohorts. The frequency of unclassifiable ILD in our cohort (25% of incident cases) is at the high end of reported values in the literature, which have ranged from 0.2 to 24% [19–22]. Some discrepancy may be attributed to referral bias, the complexity of cases assessed at our recruitment sites, or the relatively short duration of follow-up without ample time for evolution of some cases to a defined ILD subtype; however, it is likely that the diagnostic criteria used in our study play an important role. We adhered to strict guideline-based criteria whenever possible, most notably requiring biopsy confirmation of idiopathic NSIP as suggested in a previous American Thoracic Society document [12]. A diagnosis of chronic HP required a minimum of two out of three domains (clinical, radiological, pathological) to be met, which likely contributed to the lower percentage seen in our cohort (7.5%). Chronic HP was a frequent leading differential diagnosis amongst those with unclassifiable ILD, suggesting the use of different diagnostic criteria for HP may impact its reported prevalence within our cohort. We also considered IPAF to be a subgroup of unclassifiable ILD given the absence of a formal recommendation for this entity to be considered a specific clinical diagnosis [14]. These approaches resulted in a high frequency of unclassifiable ILD in our cohort, as well as a very low frequency of idiopathic NSIP (0.9%). In contrast, idiopathic NSIP was diagnosed in 8.5% of patients in an Indian registry that did not require a SLB for diagnosis [19], and was a frequent differential diagnosis in our patients with unclassifiable ILD. It is therefore unclear if we have underestimated the prevalence of idiopathic NSIP or if it has been over-diagnosed in other cohorts. Regardless, the high frequency of unclassifiable cases after a SLB and multidisciplinary discussion in our cohort highlights the limitations of current diagnostic algorithms and the challenges clinicians face when determining ILD subtype [13]. The extent to which this influences individual patient management and outcomes requires further study.

Many observational studies have suggested links between various environmental exposures and multiple subtypes of ILD. The strength of association varies between exposure and disease, with strong etiologic links established for diseases such as HP, asbestosis, and silicosis [8, 23, 24]. While direct causal links are difficult to establish, exposures such as cigarette smoke, metal dusts, and farming have been frequently associated with IPF [8]. Patient-reported exposures in our cohort were common in all diagnoses, with organic exposures more frequently endorsed than inorganic ones. Not surprisingly, organic exposures were most frequent in chronic HP. Organic exposures were also reported by over 50% of patients with IPF, which is higher than reported in other registries [4], and possibly related to our broad definition of exposures that included items such as down feathers, musty smell, and standing water. One-quarter of IPF patients also endorsed an inorganic exposure, which is similar to the reported frequency in the German INSIGHTS-IPF registry [4].

The high frequency and importance of comorbidities in patients with ILD is increasingly recognized. Studies suggest increased healthcare costs and physician visits in ILD patients compared to matched controls [2, 25], with most studies focusing on patients with IPF and little information available for patients with other ILD subtypes. Patient-reported comorbidities were common in our cohort, with GERD (26.6%), COPD (23.1%), diabetes (15.2%), and OSA (14.5%) most frequently endorsed. The prevalence of comorbidities in patients with IPF was similar to previously described cohorts [4]. We found that the frequency of comorbidities was lower in sarcoidosis compared to other diagnoses, likely reflecting the younger age of this group. The CCI was highest in CTD-ILD, reflecting the additional point that these patients received for a diagnosis of CTD. Removing CTD from the index, mean CCI was lowest in patients with CTD-ILD, likely reflecting the younger age of these patients. Although the CCI is a validated tool that predicts 1-year mortality in older adults [15], it has never been specifically validated in patients with ILD. We chose to include the CCI in CARE-PF given the lack of an alternative validated comorbidity index for ILD at the time of study design, it’s extensive use and validation in other chronic diseases [26–28] and validated use in health services research [29–31]. However, the CCI had a very narrow distribution across our cohort of fibrotic ILD, suggesting it may be a poor discriminator in assessing the impact of comorbidities on patients with ILD. Additionally, the CCI does not capture all comorbidities that are relevant in ILD such as GERD and OSA. Furthermore, the CCI fails to account for functional limitation, which is increasingly recognized as an important component of frailty and a strong predictor of mortality in many chronic diseases [32–35].

Our study has several limitations. First, CARE-PF is a prospective multicenter cohort of patients seen at specialized ILD centers and may not be representative of a general ILD population. While each site is a regional referral center and accepts patients from wide geographic areas, long travel times are a logistical barrier to many patients in northern and rural Canada. Second, we standardized the diagnostic approach for some ILDs that lack diagnostic criteria in order to maintain consistency across enrollment sites; however, our pre-specified approach may be different than that taken in some settings. Third, some of the data were based on patient report, including exposure history and some comorbidities, which could result in over- or under-reporting. Lastly, we were unable to assess longitudinal data (e.g., lung function decline, hospitalizations, mortality) due to the short duration of follow-up. Further analyses in this regard are planned for future studies. Despite these limitations we were able to characterize a large cohort of fibrotic ILD patients, expanding on previous research which has primarily focused on IPF.

## Conclusions

In summary, we report the baseline characteristics of CARE-PF, a large prospective cohort of Canadian patients with fibrotic ILD. Consistent with other large multicenter cohorts, we report a high prevalence of IPF and CTD-ILD, but with high prevalence of unclassifiable ILD and low prevalence of idiopathic NSIP when using standardized and rigorous diagnostic criteria. We also show a high frequency of patient-reported exposures across all major ILD subtypes, suggesting the limitation of using patient self-report for these ILD risk factors. Finally, we demonstrate the uncertain value of the CCI in fibrotic ILD given the limited distribution and range of abnormal values. Long-term follow-up of these patients will allow additional longitudinal analyses to gain further insights into ILD phenotypes and outcomes.

## Supplementary information

**Additional file 1: Table S1.** Exposures captured in the CAnadian REgistry for Pulmonary Fibrosis.

**Additional file 2: Table S2.** Components of the Charlson Comorbidity Index.

**Additional file 3: Table S3.** Proportion of ILD diagnoses for incident, prevalent and overall cases.

**Additional file 4: Table S4.** Baseline characteristics of incident cases.

## Data Availability

The data that support the findings of this study are available from the CARE-PF investigators but restrictions apply to the availability of these data, which were used under license for the current study, and so are not publicly available. Data are however available from the authors upon reasonable request and with permission of the CARE-PF Steering Committee.

## Abbreviations

CARE-PF: CAnadian REgistry for Pulmonary Fibrosis
CCI: Charlson Comorbidity Index
COPD: Chronic obstructive pulmonary disease
CTD: Connective tissue disease
CTD-ILD: CTD-associated ILD
GERD: Gastroesophageal reflux disease
HP: Hypersensitivity pneumonitis
IIP: Idiopathic interstitial pneumonia
ILD: Interstitial lung disease
IPAF: Interstitial pneumonia with autoimmune features
NSIP: Non-specific interstitial pneumonia
OSA: Obstructive sleep apnea
SLB: Surgical lung biopsy
