# Supplementary material for: Baseline characteristics and comorbidities in the CAnadian REgistry for Pulmonary Fibrosis
Source: BMC Pulm Med. 2019 Nov 27;19:223. doi: 10.1186/s12890-019-0986-4 (PMC6880596; doi:10.1186/s12890-019-0986-4)
Supplement: Supplementary file 1 — Additional file 1: Table S1. Exposures captured in the CAnadian REgistry for Pulmonary Fibrosis. [file 12890_2019_986_MOESM1_ESM.docx]

**Table S1**. Exposures captured in the CAnadian REgistry for Pulmonary Fibrosis.

| **Exposure Category** | **Exposure Question** |
| --- | --- |
| **Environmental exposures**  Answer “Yes” if you were REGULARLY OR REPEATEDLY EXPOSED to any of the following in the THREE YEARS BEFORE YOUR BREATHING PROBLEM STARTED | Indoor hot tub or steam sauna |
|  | Water leaks (e.g. leaky appliances) or mold/mildew in the home |
|  | Down pillows or comforters |
|  | Do you own or are regularly exposed to pigeons, parakeets or other birds |
|  | Does your house or office smell musty? |
|  | Has there been a history of flooding? |
|  | Do you have standing water in the home (e.g. humidifier, fish tank, water pans in appliances, swamp cooler) |
|  | Do you work with potting soils or compost on a regular basis? |
|  | Do you live or work on a farm? |
| **Occupational exposures**  Answer “Yes” if you have ever worked as one of the following. | Pottery worker |
|  | Pipe worker/plumber |
|  | Insulation worker (pipe/boiler, bulkhead linings, filler, grouting) |
|  | Farmer |
|  | Sandblaster |
|  | Rock miner or mica worker |
|  | Talc worker |
|  | Beryllium worker |
|  | Aluminum worker |
|  | Plastic worker |
|  | Railroad worker |
|  | Longshoreman |
|  | Smelter/Foundry worker |
|  | Welder |
|  | Road builder/tunnel construction worker |
|  | Cement/cement product worker |
|  | Automotive product worker (brake linings, gaskets, clutch plates) |
| **Medication and treatment exposures**  Please indicate whether you currently take the treatment, took the treatment in the past, or have never received the treatment. | Amiodarone (Cordarone®) |
|  | Nitrofurantoin (Macrobid®, Macrodantin®) |
|  | Methotrexate (Folex®, Rheumatrex®) |
|  | Radiation therapy to the chest |
|  | Cancer chemotherapy (e.g. bleomycin) |
|  | Biologic (specify if known): |
