# Supplementary material for: Baseline characteristics and comorbidities in the CAnadian REgistry for Pulmonary Fibrosis
Source: BMC Pulm Med. 2019 Nov 27;19:223. doi: 10.1186/s12890-019-0986-4 (PMC6880596; doi:10.1186/s12890-019-0986-4)
Supplement: Supplementary file 2 — Additional file 2: Table S2. Components of the Charlson Comorbidity Index. [file 12890_2019_986_MOESM2_ESM.docx]

**Table S2**. Components of the Charlson Comorbidity Index.

| **Comorbidity** | **Assigned weight** |
| --- | --- |
| Myocardial infarct | 1 |
| Congestive heart failure | 1 |
| Peripheral vascular disease | 1 |
| Cerebrovascular disease | 1 |
| Dementia | 1 |
| Chronic pulmonary disease | 1 |
| Connective tissue disease | 1 |
| Ulcer disease | 1 |
| Mild liver disease | 1 |
| Diabetes | 1 |
| Hemiplegia | 2 |
| Moderate or severe renal disease | 2 |
| Diabetes with end organ damage | 2 |
| Any tumor | 2 |
| Leukemia | 2 |
| Lymphoma | 2 |
| Moderate or severe liver disease | 3 |
| Metastatic solid tumor | 6 |
| AIDS | 6 |

1-year mortality based on the Charlson Comorbidity Index in the original

derivation population for patients discharged from hospital after admission

for a ‘mild illness’ was 7% for a score of 0, 16% for a score of 1-2, 41% for a

score of 3-4 and 64% for a score of > 5.(1)
