# Supplementary material for: Baseline characteristics and comorbidities in the CAnadian REgistry for Pulmonary Fibrosis
Source: BMC Pulm Med. 2019 Nov 27;19:223. doi: 10.1186/s12890-019-0986-4 (PMC6880596; doi:10.1186/s12890-019-0986-4)
Supplement: Supplementary file 3 — Additional file 3: Table S3. Proportion of ILD diagnoses for incident, prevalent and overall cases. [file 12890_2019_986_MOESM3_ESM.docx]

**Table S3.** Proportion of ILD diagnoses for incident, prevalent and overall cases.

| **Diagnosis** | **Incident cases (n=961)** | **Prevalent cases**  **(n=324)** | **Overall cohort (n=1285)** |
| --- | --- | --- | --- |
| IPF | 253 (26.3) | 64 (19.8) | 317 (24.7) |
| Non-IPF IIP | 28 (2.9) | 11 (3.4) | 39 (3.0) |
| Chronic HP | 69 (7.2) | 28 (8.6) | 97 (7.5) |
| Sarcoid | 36 (3.7) | 5 (1.4) | 41 (3.2) |
| CTD-ILD | 278 (28.9) | 150 (46.3) | 428 (33.3) |
| Unclassifiable ILD | 238 (24.8) | 48 (14.8) | 286 (22.3) |
| Other ILD | 59 (6.1) | 18 (5.6) | 77 (6.0) |

Data shown are number (percent).

Other ILD includes: Vasculitis, diffuse alveolar hemorrhage, drug related, pneumoconiosis, post-acute respiratory disease syndrome, aspiration, eosinophilic pneumonia, pleuroparenchymal fibroelastosis, lymphangioleiomyomatosis, Langerhan’s cell histiocytosis, neuroendocrine cell hyperplasia, pulmonary alveolar proteinosis.

Abbreviations: CTD-ILD, connective tissue disease-associated ILD; HP, hypersensitivity pneumonitis; IIP, idiopathic interstitial pneumonia; ILD, interstitial lung disease; IPF, idiopathic pulmonary fibrosis.
