# Supplementary material for: Baseline characteristics and comorbidities in the CAnadian REgistry for Pulmonary Fibrosis
Source: BMC Pulm Med. 2019 Nov 27;19:223. doi: 10.1186/s12890-019-0986-4 (PMC6880596; doi:10.1186/s12890-019-0986-4)
Supplement: Supplementary file 4 — Additional file 4: Table S4. Baseline characteristics of incident cases. [file 12890_2019_986_MOESM4_ESM.docx]

**Table S4.** Baseline characteristics of incident cases.

| **Characteristic** | **Full cohort**  **(n= 961)** | **IPF**  **(n= 253)** | **Non-IPF IIP (n= 28)** | **HP**  **(n= 69)** | **Sarcoid**  **(n= 36)** | **CTD**  **(n= 278)** | **Unclassifiable (n= 238)** | **Other ILD**  **(n= 59)** |
| --- | --- | --- | --- | --- | --- | --- | --- | --- |
| Age | 65.2+/-11.8 | 71.2+/-8.5 | 59.9+/-10.7 | 62.8+/-10.3 | 56.3+/-12.0 | 61.0+/-11.7 | 67.5+/-10.6 | 60.6+/-15.9 |
| Male sex | 493 (51.4) | 185 (73.1) | 15 (53.6) | 34 (49.3) | 18 (50.0) | 85 (30.6) | 126 (53.2) | 30 (50.9) |
| BMI | 29.0+/-5.9 | 28.5+/-4.7 | 30.3+/-6.3 | 30.7+/-6.9 | 29.0+/-6.2 | 27.6+/-6.1 | 30.3+/-5.7 | 28.9+/-6.7 |
| FVC percent | 74.4+/-20.4 | 73.1+/-19.8 | 72.9+/-19.9 | 66.2+/-18.5 | 85.9+/-16.2 | 73.6+/-19.7 | 76.2+/-21.3 | 79.5+/-22.1 |
| DLCO percent | 58.0+/-20.3 | 50.2+/-16.6 | 57.6+/-20.4 | 53.1+/-18.5 | 81.1+/-18.3 | 58.0+/-19.6 | 60.3+/-19.9 | 71.3+/-24.0 |
| Oxygen | 194 (21.9) | 78 (32.8) | 7 (25.9) | 21 (33.9) | 2 (5.7) | 41 (16.1) | 33 (15.4) | 12 (21.4) |
| SLB | 185 (19.3) | 31 (12.3) | 12 (42.7) | 39 (56.5) | 14 (38.9) | 24 (8.6) | 51 (21.4) | 14 (23.7) |

* Data shown are mean+/–standard deviation, median (interquartile range) or number (percent).

Other ILD includes: Vasculitis, diffuse alveolar hemorrhage, drug related, pneumoconiosis, post-acute respiratory disease syndrome, aspiration, eosinophilic pneumonia, pleuroparenchymal fibroelastosis, lymphangioleiomyomatosis, Langerhan’s cell histiocytosis, neuroendocrine cell hyperplasia, pulmonary alveolar proteinosis.

Abbreviations: BMI, body mass index; CTD-ILD, connective tissue disease-associated ILD; DLCO, diffusing capacity of the lung for carbon monoxide; FVC, forced vital capacity; HP, hypersensitivity pneumonitis; IIP, idiopathic interstitial pneumonia; ILD, interstitial lung disease; IPF, idiopathic pulmonary fibrosis; SLB, surgical lung biopsy.
